# An experimental study of the effects of bacteria on asphaltene adsorption and wettability alteration of dolomite and quartz

**DOI:** 10.1038/s41598-023-48680-7

**Published:** 2023-12-06

**Authors:** Younes Soleimani, Mohammad-Reza Mohammadi, Mahin Schaffie, Reza Zabihi, Mohammad Ranjbar

**Affiliations:** 1 Department of Petroleum Engineering, Shahid Bahonar University of Kerman, Kerman, Iran; 2 Department of Mining Engineering, Shahid Bahonar University of Kerman, Kerman, Iran

**Keywords:** Energy science and technology, Engineering

## Abstract

The adsorption of asphaltene on the rock surface and the changes in its wettability are very relevant issues in flow assurance and oil recovery studies, and for carbonate reservoirs, they are even more important. During microbial enhanced oil recovery (MEOR) processes, wettability alteration is considered a crucial mechanism leading to improved oil recovery. Therefore, it is essential to understand the mechanisms of surface wettability changes by bacteria and biosurfactants and find new and reliable methods to prevent asphaltene adsorption. Hence, the main aim of this research was to investigate the effect of a mixture of thiobacillus thiooxidans and thiobacillus ferooxidans microorganisms with an optimum effective temperature of around 30 °C (referred to as mesophilic bacteria), as well as a mixture of two moderate thermophiles Sulfobacillus thermosulfidooxidans for operating temperatures around 50 °C (referred to as moderately thermophilic bacteria) on the adsorption of asphaltene samples isolated from two different crude oils onto main reservoir minerals (i.e., quartz and dolomite). The results indicated that after two weeks of mineral aging in moderate thermophilic bacteria, the adsorption of asphaltene on both minerals increased between 180 and 290%. Fourier-transform infrared spectroscopy (FTIR) analysis for quartz and dolomite samples demonstrated that after aging in bacterial solution, bonds related to the adsorption of bacterial cells and biosurfactant production appear, which are the main factors of change in wettability. Alteration in wettability towards hydrophilicity expands hydrogen bonds on the surface, thus improving asphaltene adsorption due to polar interaction. Asphaltene 1 changed the contact angle of dolomite from 53.85° to 90.51° and asphaltene 2 from 53.85° to 100.41°. However, both strains of bacteria caused a strong water-wetting effect on the dolomite rock samples. The influence of moderate thermophilic bacteria on surface wettability is more significant than that of mesophilic bacteria, which may be caused by the high protein content of these bacteria, which expands hydrogen bonding with the surface. Adsorption of asphaltenes on dolomite rocks previously aged with bacteria showed that the wetted rock samples retained their water-wet state. This study highlights the dual impact of the used microorganisms. On one hand, they significantly reduce contact angles and shift wettability towards a strongly water-wet condition, a crucial positive factor for MEOR. On the other hand, these microorganisms can elevate the adsorption of asphaltenes on reservoir rock minerals, posing a potential challenge in the form of formation damage, particularly in low-permeability reservoirs.

## Introduction

Asphaltenes are the heaviest, most polar, densest, and surface-active fraction of crude oil. They are defined as complex molecular structures that include aromatic rings, alkyl chains, and aliphatic side chains along with functional groups and heteroatoms^1–3^. The considerable reactions and interactions of the asphaltene molecule rise from heteroatoms that construct the polar structure of asphaltene and can form aggregate^4, 5^. Asphaltenes are in equilibrium with other crude oil compounds under pristine reservoir conditions. Any change in equilibrium conditions, such as pressure, oil composition, or, temperature can lead to self-aggregation and precipitation of asphaltene particles from crude oil^1, 6^. The deposition of asphaltene bears profound consequences on the petrophysical properties of reservoir rock, pipes, and surface facilities. Adsorption of asphaltene on the surface of pores, flow throat blockage, and changes in rock wettability can cause problems such as phase and flow behavior changes, reduction of production efficiency in exploitation and enhanced oil recovery (EOR) operations^7–9^. Understanding the adsorption behavior of asphaltenes on the rock surface in porous media can provide a better solution to treat these deposits^10^. The adsorption behavior of asphaltene on different surfaces depends on the structural properties of asphaltene, their concentration, gas-oil ratio, water, the surface morphology of adsorbents, wettability, and operating temperature and pressure conditions^11–14^. In recent decades, investigators have focused on the critical phenomenon of asphaltene precipitation and its adsorption on various surfaces^14–26^. One of the effective methods to solve the problem of asphaltene flocculation and precipitation is utilizing adsorbents^27, 28^. Adsorbents from micro to nanoparticle sizes can effectively remove asphaltenes from oil^17, 29^. Many researchers investigated the surface adsorption of asphaltene on different adsorbents in the presence and absence of water. They figured that asphaltene adsorption on the surfaces decreases in the presence of water^30–34^. Almost most studies have shown that the model of asphaltene adsorption on surfaces depends on asphaltene concentration. As the initial concentration of asphaltene increases, the amount of asphaltene adsorption on the adsorbents increases^2, 12, 35^. The role of heteroatoms in the uptake of asphaltenes by quartz adsorbents has shown that sometimes the nitrogen content is independently effective^36^, and sometimes the nitrogen content and the asphaltene polarity have a synergistic effect on the amount of asphaltene adsorbed on the adsorbents^35^. A recent study has indicated that the process of asphaltene adsorption on SiO_2_ nanoparticles is physical in nature, and the interaction forces between the particles are dominant in the process^37^. Regarding the adsorption of asphaltenes on dolomite, the total amount of nitrogen and sulfur along with the aromaticity of asphaltenes play an important role in the adsorption process^38^.

Reservoir rock wettability is a fundamental concept in reservoir engineering and has been the issue of numerous investigations over the years because of its significant impact on oil recovery. The primary role of wettability in a reservoir is to determine the location and distribution of reservoir fluids, which affects the relative permeability of the reservoir fluid and determines recovery efficiency. Crude oil wets rocks through various mechanisms, including acid–base interactions, ionic bonding, surface precipitation, and polar interactions between surfaces and oil polar groups in the absence of water^39–42^. The wettability of a reservoir is intensely related to the adsorption amount of the most polar compounds present in crude oil, especially the asphaltene and resin fractions^39^. Asphaltenes are predominantly deposited on rock surfaces non-uniformly, relying on the shape of the pores, mineralogy, and surface roughness, and cause complete or partial changes in rock wettability^9^. Many researchers investigated the effect of asphaltene and other crude oil compounds on the wettability of minerals. They showed that organic precipitation (especially asphaltene) is the main factor in altering the wettability of minerals from water-wet to oil-wet. Aging time, initial water saturation, crude oil composition, aging temperature, type of solvent, and asphaltene compounds influence wettability changes^43–50^. The polarization interaction mechanism is found to be the primary method for changing the wettability of calcite and dolomite samples by asphaltene and resin^49^. Moreover, heteroatoms and aromatic rings as functional groups can affect the process of asphaltene accumulation and adsorption on the silica surface and make its surfaces oil-wet^51^. The impact of microorganisms on wettability has also been investigated and it has been found that the initial wetting conditions, surface properties, types of microorganisms, and their metabolic products are the influencing parameters. Adhesion of bacteria to the surface, biofilm formation, and adsorption of ingredients from the bacterial solution, and biosurfactants are the suggested mechanisms for changing wettability during microbial enhanced oil recovery (MEOR) processes. However, more than one mechanism can work simultaneously^52^. Studies of alteration in wettability due to the effect of bacteria and biosurfactants on rock surfaces and pores indicate that wettability can alter to neutral or extremely water-wet^53–55^. An increase in microbial concentration has led to a decrease in the contact angle to a certain extent, and in the presence of bacteria, crude oil containing low asphaltene concentration has created a more stable contact angle^56^. In addition, it has been clarified that bacteria play a pivotal role in reducing the interfacial tension of reservoir fluids drastically^57, 58^, thereby playing a crucial role in enhancing oil recovery. As far as our observations show, little attention has been paid to the effect of bacteria on the adsorption of asphaltene onto the main minerals found in reservoir rocks. Using new effective methods to prevent the precipitation and adsorption of asphaltene and the consequent damage has become imperative. Moreover, it is usually stated that wettability alteration of carbonate reservoirs is somehow more important than sandstone reservoirs, because their nature promotes the adsorption of organic components of crude oil onto the rock surface, making oil recovery a challenge even with the interventions of various chemical EOR techniques^59^. Hence, the aforementioned challenges present an opportunity for further investigation.

In this research, the effect of two bacteria samples, isolated from copper complex sewerage, is investigated on the adsorption of asphaltenes, isolated from two different crude oil samples, on quartz and dolomite in a two-phase system (solid adsorbent + asphaltene in toluene solution). X-ray fluorescence spectroscopy (XRF) and Fourier transform infrared spectroscopy (FTIR) techniques are used to analyze the adsorbent samples. Also, adsorption experiments are conducted using a UV–VIS spectrophotometer. In addition, FTIR analyses are performed to identify functional groups of quartz and dolomite adsorbents before adsorption, after aging in bacteria, and after asphaltene adsorption. Moreover, in wettability tests, the influences of asphaltene adsorption, bacteria, and asphaltene adsorption after the impact of bacteria on the wettability change of dolomite are evaluated through contact angle analysis. Eventually, this study brings into focus the pivotal role of the used microorganisms, elucidating their impact on EOR process and acknowledging the potential challenge they pose in the form of formation damage.

## Materials and methods

**Figure 1:**
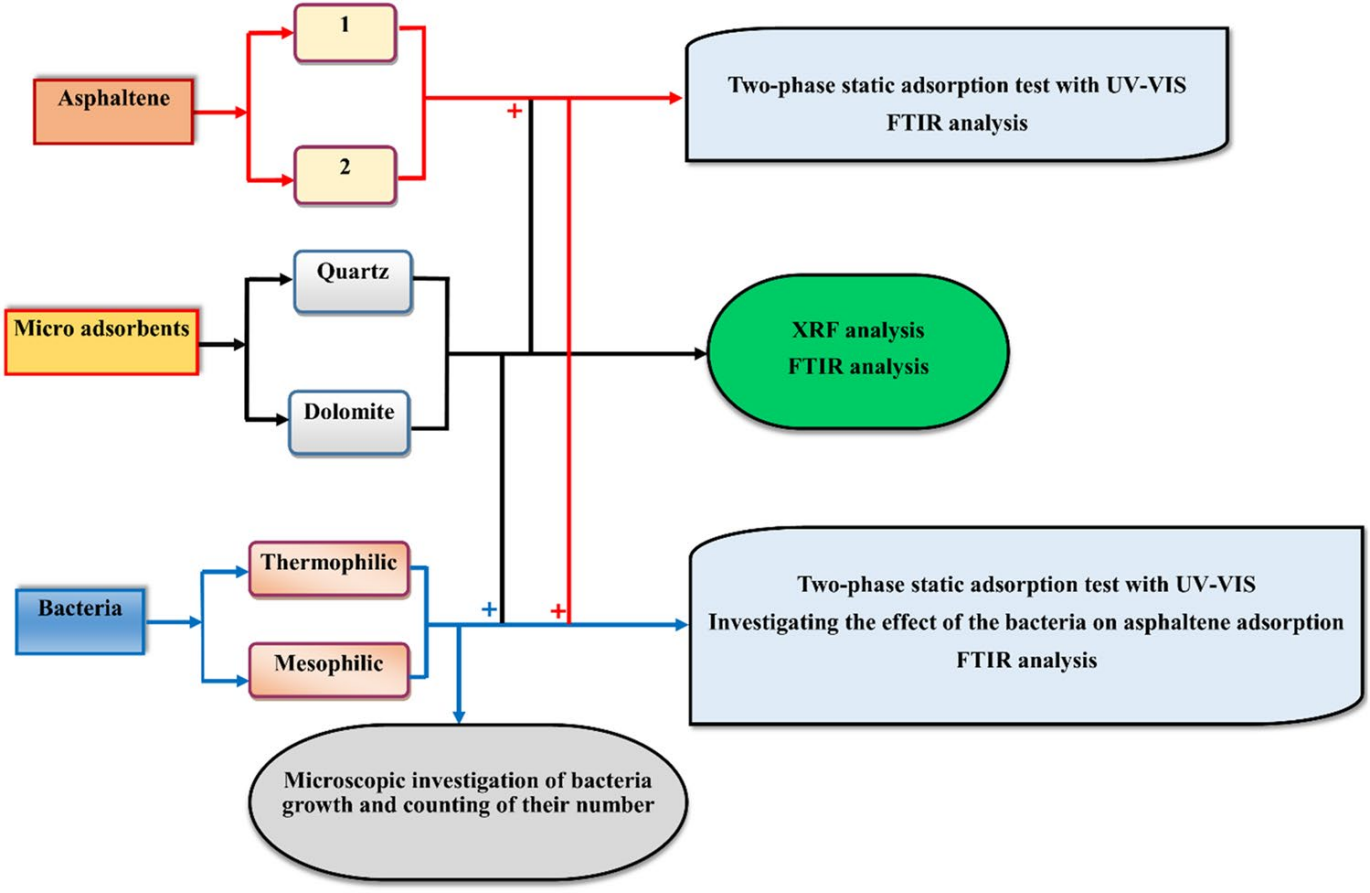
Workflow of the adsorption experiments.

The schematic of the steps taken in this study is depicted in Fig. 1. In this study, asphaltene adsorption tests were performed with three repetitions to ensure reliability and accuracy.

### Materials and sample preparation

#### Adsorbent

This research used quartz and dolomite, the main minerals of sandstone and carbonate formations, as adsorbents. Using (PHILIPS, PW1410, Netherland), XRF analysis was performed to determine the composition of minerals (Table 1); Quartz and dolomite were crushed to a particle size range of 75–125 µm and dubbed as micro quartz and micro dolomite. High loss on ignition (LOI) content of 43% in the dolomite sample may be attributed to the presence of impurities or secondary minerals with volatile components. Dolomite-rich formations often include variable amounts of clay minerals or organic matter, which can contribute to elevated LOI values. Additionally, the heating conditions during LOI determination might have triggered hydration or decomposition reactions, releasing volatile gases^60^. This finding aligns with prior studies that highlight the influences of impurities and/or releasing volatile gases during heating on high LOI content in dolomite or carbonate-rich samples^60–62^.

**Table 1:** XRF analysis of quartz and dolomite.

Component (wt%)	Quartz	Dolomite^30^
SiO_2_	95.43	1.13
A_l2_O_3_	0.21	0.04
BaO	< 0.01	0.01
CaO	0.31	36.02
Fetotal	1.84	6.54
K_2_O	1.17	0.01
MgO	0.1	12.47
MnO	< 0.01	0.62
Na_2_O	< 0.01	0.09
P	< 0.01	0.03
S	0.1	< 0.05
TiO_2_	0.13	< 0.01
Cr_2_O_3_	< 0.01	< 0.01
Cu	< 0.01	< 0.01
Pb	< 0.01	< 0.01
Zn	< 0.01	< 0.01
LOI*	< 0.01	43.04

*Loss on ignition.

#### Asphaltene

The used asphaltenes are related to two different oil samples from different oil fields located in the southwest of Iran, which were separated using n-heptane precipitant based on ASTM standard separation method (D2007-80)^30, 63^. Oil 1, from which asphaltene 1 was extracted, has an API gravity of 19° and an asphaltene content of 13.1% by mass. On the other hand, Oil 2, from which asphaltene 2 was extracted, exhibits an API gravity of 32.5° and an asphaltene content of 1.3% by mass. The composition and structure of these asphaltenes were investigated completely using various analyses, including field emission scanning electron microscopy analysis, dynamic light scattering analysis, FTIR analysis, and elemental analysis, which can be found in the literature^30, 38^.

#### Rock samples and fluid

Investigations were carried out to analyze the surface wettability of dolomite rocks. The rocks were cut into tiny slabs; each slab’s height, width, and length were 0.5, 2.5, and 2.5 cm, respectively. The surface of the samples was completely polished to remove the effect of mineral surface roughness on contact angle measurement. The slabs were located in a Soxhlet chamber, including 500 ml of toluene, and cleaned for 2–4 h. Then, the samples were kept in the oven at 50 °C for one hour. Then the samples were cleaned with double distilled water for 24 h and placed in an oven at 50 °C for 24 hours^49^. This approach was utilized to reduce possible errors, clean the surface, and avoid the effect of organic contaminants on the surface in the wetting properties measurements.

In this study, Persian Gulf water was utilized to assess the influence of wettability in the contact angle experiments. The pH of Persian Gulf water is approximately 8 and it comprises ions such as *Cl*^*−*^, ${HCO}_{3}^{2-}$, ${SO}_{4}^{2-}$, *Na*^+^, *K*^+^, *Mg*^*2*+^, and *Ca*^*2*+^ with a total dissolved solids content of around 42 g/l. Also, the density of this water is approximately 1.027 g/ml^64, 65^.

#### Microorganism

In this research, a mixture of thiobacillus thiooxidans and thiobacillus ferooxidans microorganisms with an optimum effective temperature of around 30 °C and a mixture of two moderate thermophiles sulfobacillus thermosulfidooxidans for operating temperatures around 50 °C were used. Both cultures were gram-negative, acidophilic, and autotrophic. These types of culture are well described in the literature^66, 67^. Here, the terms "mesophilic" and "moderate thermophilic" were employed to differentiate between the mentioned microorganisms. Both types were isolated from the sewerage of the Iranian copper facility. Since the microorganisms were necessarily aerobic, they were assigned to the obligate aerobic category^57^. Here, 9k medium was renowned for activating, accommodating, and growing such microorganisms^57, 68^.

### Methods

#### Preparation of asphaltene/toluene solution

The 200 ppm asphaltene solution in toluene was prepared by dissolving 0.2g of dried asphaltenes in 1 L of toluene. In order to determine the relationship between the concentration and adsorption of asphaltenes/toluene, firstly, the adsorption of several certain concentrations of asphaltenes/toluene was measured by a UV–VIS spectrophotometer (Cary 100, Germany) at a fixed wavelength of 410 nm. This wavelength was considered appropriate and within the absorbance threshold of the instrument as it had reasonable sensitivity for assessing asphaltene concentrations^16, 17, 69^. Furthermore, the unknown concentrations of asphaltene were determined using this calibration curve.

#### Microscopic examination of microorganisms

In order to inspect the growth of bacteria, images were taken before growing in the culture medium. Then, 9k medium^57, 68^ was utilized to grow bacteria in the shaker incubator (Kuhner shaker x, Germany) at a temperature of 30 or 50 ℃ and 91 rpm. After the bacteria entirely grew and were in the stationary growth phase, the number of bacteria was counted using a microscope (Nikon TE2000-U, Japan) in order to determine their concentration by employing the Petroff-Hausser counting chamber. Finally, bacteria were applied in the experiments.

#### Static adsorption tests

##### Adsorption of asphaltene in different amounts of adsorbent

A specific amount of adsorbent is added to a specific volume of an asphaltene/toluene solution with a specific concentration. After a certain time (equilibration time), the residual asphaltene concentration was determined by employing a UV–VIS spectrophotometer and a calibration curve. After a specific time, asphaltene adsorption on the adsorbent reaches a maximum point. Any increase in duration beyond that point leads to a steady state of adsorption known as equilibrium time^35^. According to the static adsorption tests that show the effect of contact time on the adsorption of asphaltenes for different adsorbents, the results indicate that the equilibrium of the process is less than 2 hours^13, 18, 30, 38^. Therefore, the equilibrium time in all experiments is 2 h for this research. The extent of asphaltenes adsorbed on the adsorbent surface was determined^17^ by Eq. (1):

1 $$q \, = \frac{{\left( {{\text{C}}_{{0}}- {\text{C}}_{{\text{e}}} } \right){\text{v}}}}{{\text{m}}}$$

Here, *q* indicates the amount of asphaltene adsorption (mg/g), *V* is the solution volume (L), *C*_*0*_ and *C*_*e*_ show the initial asphaltene concentration and equilibrium concentration after asphaltene adsorption (mg/L), subsequently, and *m* denotes the mass of the adsorbent (g). Also, the amount of asphaltene removed from the asphaltene/toluene solution was computed^17^ by Eq. (2):

2 $$Asphaltene \, \;removal \, \;\left( \% \right) \, = { }\frac{{{\text{C}}_{{0}} -{\text{C}}_{{\text{e}}} }}{{{\text{C}}_{{0}} }} \times 100$$

In this experiment, different mass amounts (0.25, 0.5, 1, and 2 g) of quartz and dolomite adsorbents were added to 10 ml of asphaltene/toluene solution with a constant concentration of 200 ppm. The mixture of adsorbent and asphaltene solution was placed in a shaker incubator (Kuhner shaker x, Germany) at 250 rpm for 2 h. The adsorption test was carried out in a two-phase system (solid adsorbent + asphaltene in toluene solution) at a temperature (50 °C) and atmospheric pressure (14.7 psia). Next, the UV–VIS spectrophotometer was used to determine the equilibrium concentration of asphaltene. Finally, the extent of asphaltene adsorption on the adsorbents was determined by applying Eq. (1).

##### Effect of bacteria on asphaltene adsorption

After the growth and activation of bacteria, samples were prepared with the ratio of minerals to bacteria solutions (1g: 10 ml). Then the samples were placed inside the shaker incubator with the same growth conditions for 1 and 2 weeks. Since the bacteria used were acidophilic and showed the best growth and activity in acidic environments, the pH of the bacterial medium was adjusted to 2 with sulphuric acid. After aging, the minerals were separated from the bacteria solution using a centrifuge (Universal), and the samples were placed in the oven for 48 h. After the minerals were dried entirely according to the same conditions as before in the first phase of the experiment (asphaltene adsorption), 10 ml of asphaltene/toluene solution with a certain concentration of 200 ppm was added to them. The samples were placed in a shaker incubator at 50 °C and 250 rpm for 2 h. Then the equilibrium concentration of asphaltene was determined using a UV–VIS spectrophotometer. Finally, asphaltene uptake by the adsorbents was determined using Eq. (1).

#### Wettability experiments

**Figure 2:**
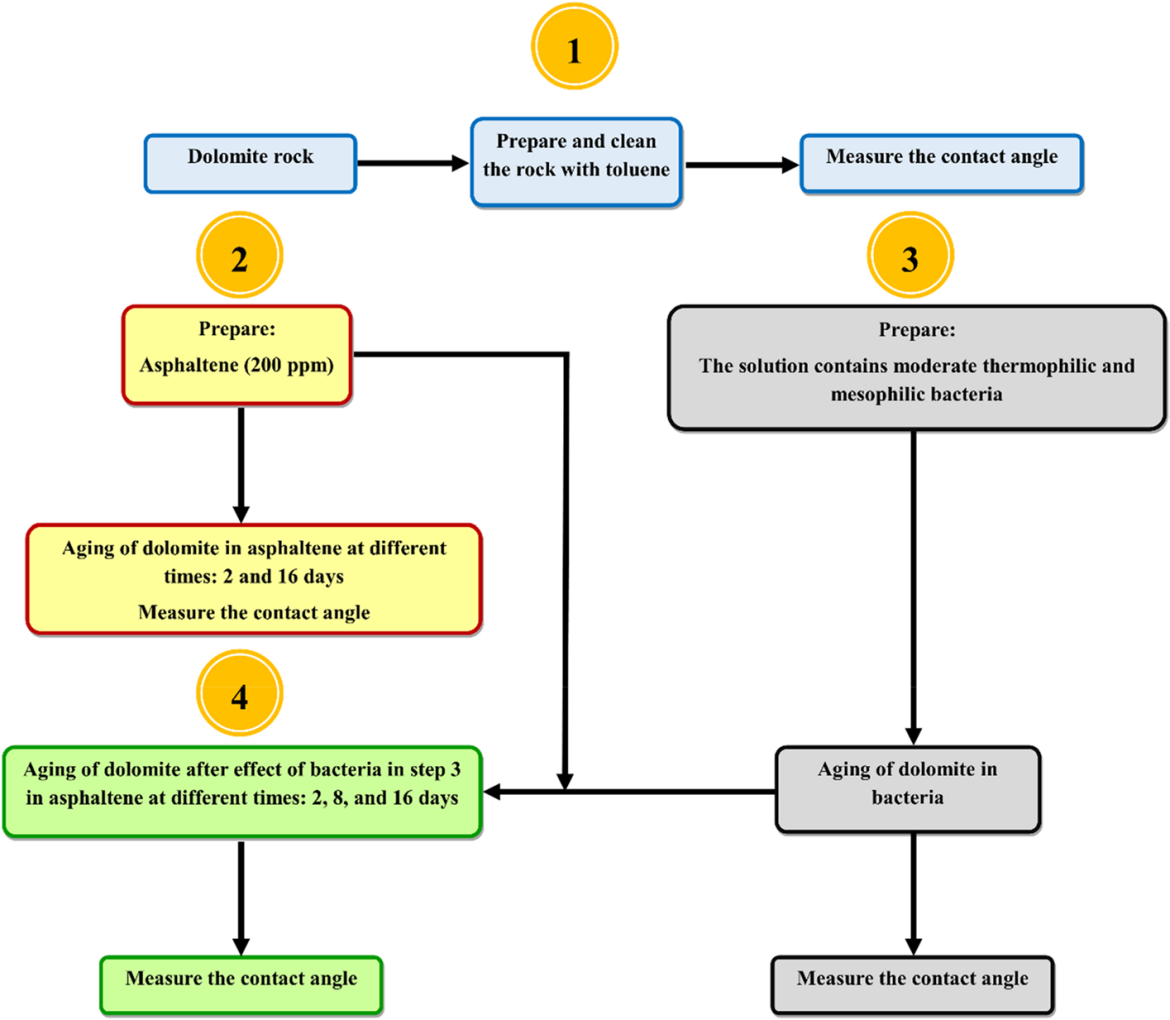
The general process of the wettability experiments.

The general process of the wettability experiments in this study is shown in Fig. 2.

##### Contact angle measurement method

The sessile drop method was used to measure the contact angle. In this procedure, to measure the contact angle, the dolomite rock samples were dried in the oven at 50 °C for 30 min and then exposed to air for 30 min to cool the surfaces. The rock samples were placed on the panel of the contact angle instrument (Krüss G2, Germany). Persian Gulf water was used as a droplet on the rock surface. A water droplet was spilled on the samples’ surface through the syringe and needle. An image of the formed droplet on the rock surface was taken with a digital camera. Finally, Image J software was utilized to obtain the contact angle and analyze the images. Measurements were repeated at least three times for each sample, and averages were reported. Also, the experiments were conducted under standard conditions of 25 °C and atmospheric pressure. According to Chillingar and Yen^70^, wettability can be categorized into three classes based on contact angles: water-wet (0–80°), neutral or intermediate wettability (80–100°), and oil-wet (100–180°).

##### Dolomite aging

In this experiment step, the contact angle of all rock samples before aging with asphaltenes and bacteria was measured. Several dolomite samples were placed directly in asphaltene solution, and the contact angles were measured at different times after aging. Subsequently, several samples were aged in moderately thermophilic and mesophilic bacteria in a pH 6.5 bacterial solution, and the contact angle was measured after 1 and 2 weeks. During aging, the bacteria regularly were investigated with a microscope. After exposure of the mineral surface to the bacteria, the samples were dried under the same conditions previously mentioned and then aged in asphaltenes. The contact angle was determined after 2, 8, and 16 days.

#### Fourier transform infrared spectroscopy (FTIR)

A (Brucker, TENSOR 27, Germany) spectrometer in the wavelength range 400–4000 cm^−1^ with 32 scans and 4 cm^−1^ resolution and potassium bromide powder (one of the most generally used alkali metal halides for FTIR analysis) was utilized to perform the FTIR analysis. Dry adsorbent, adsorbent after aging in bacteria, and the adsorbent after asphaltene adsorption were mixed with potassium bromide (KBr) powder in a ratio of 1:100. The mixed samples were then compressed to gain a transparent tablet. The moisture content of the formed tablet was removed using an oven to enhance the spectral results. This procedure is one of the qualitative techniques for determining chemical structures and species^38, 49, 71^.

## Results and discussion

### Asphaltenes characterization

Table 2 represents the elemental analysis of two asphaltene samples used in this study, as reported in the literature^30, 35^. A literature review indicates that the H/C molar ratio of two asphaltene samples is similar to that of other asphaltenes^4, 72, 73^. An asphaltene sample with a lower H/C ratio exhibits a more aromatic nature^18, 74^. Table 3 shows different indexes that reveal the structural characteristics of asphaltene samples. These indexes are obtained based on the area under peaks in the FTIR analysis and reported in the literature^30, 35^. It can be seen from Tables 2 and 3 that both asphaltene samples have high aromatic content, which may enhance the association between asphaltene and the adsorbent and increase asphaltene adsorption. On the other hand, polarity depends on the presence of heteroatoms and metals within the composition of the asphaltenes^4, 75^. Taking into account the heteroatom content as a marker of polarity, as proposed in the literature^4^, the results reported in Table 2 show that asphaltene 1 exhibits higher polarity in comparison to asphaltene 2. Also, considering heteroatoms and O/C molar ratios reported in Table 2, there is a slight correlation between the O/C molar ratio and polarity in asphaltenes. Higher O/C ratios may indicate a higher proportion of oxygen-containing functional groups within the asphaltene molecule. The presence of oxygen-containing groups often increases the polarity of a molecule, as these groups can participate in hydrogen bonding and other polar interactions. It has been found that both polarity and aromaticity play an essential role in asphaltene instability and aggregation behavior^4, 75^. However, asphaltenes, known for their intricate molecular structures and high molecular weights, encompass a variety of heteroatoms and metals. Discussing the relationship between polarity and O/C ratios requires a broader investigation involving a larger number of asphaltenes. However, this goal falls beyond the scope of the present study.

**Table 2:** Elemental analysis of asphaltenes.

	C, %	H, %	S, %	N, %	O, %	Heteroatoms, %	H/C Molar ratio	O/C Molar ratio
Asphaltene 1^35^	80.62	8.64	5	1.26	4.48	10.74	1.276	0.0417
Asphaltene 2^30^	82.58	8.35	3.07	1.47	4.53	9.07	1.204	0.0412

**Table 3:** Structural indexes of asphaltenes based on FTIR analysis.

Asphaltene	Sulphoxide index	Aliphatic index	Aromatic index
Asphaltene 1^35^	0.066	0.047	1.315
Asphaltene 2^30^	0.1177	0.0105	1.35

### Microscopic investigation of microorganisms

**Figure 3:**
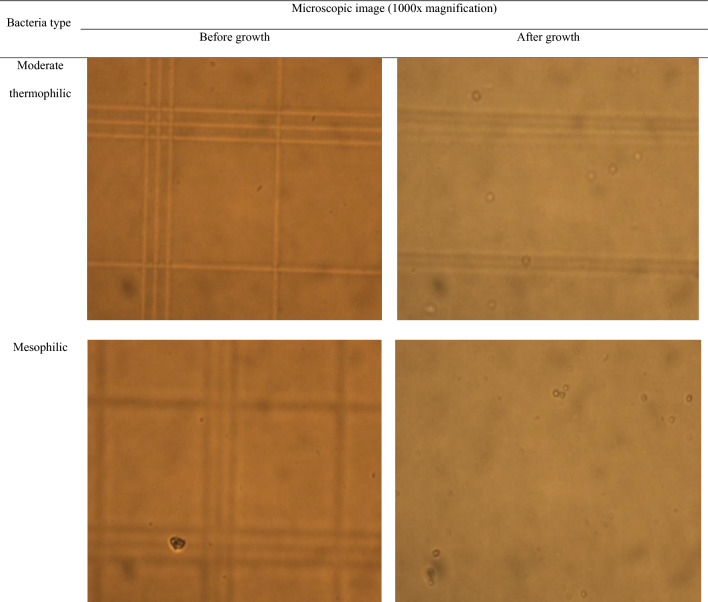
Microscopic images of microorganisms before and after activation.

Microscopic images with a magnification of 1000× before and after the activation of microorganisms are shown in Fig. 3. After activating microorganisms in the 9k medium, the Petroff-Hassuer counting chamber was utilized to check their number. This technique used a glass slide chamber with a certain depth and a grid. Table 4 shows the results of counting bacteria.

**Table 4:** Counting the number of bacteria by Petroff-Hausser chamber.

Bacteria type	Number of bacteria/cm^3^
Moderate thermophilic	10^7^ × 1.25
Mesophilic	10^7^ × 1.375

### FTIR spectroscopic analysis

**Figure 4:**
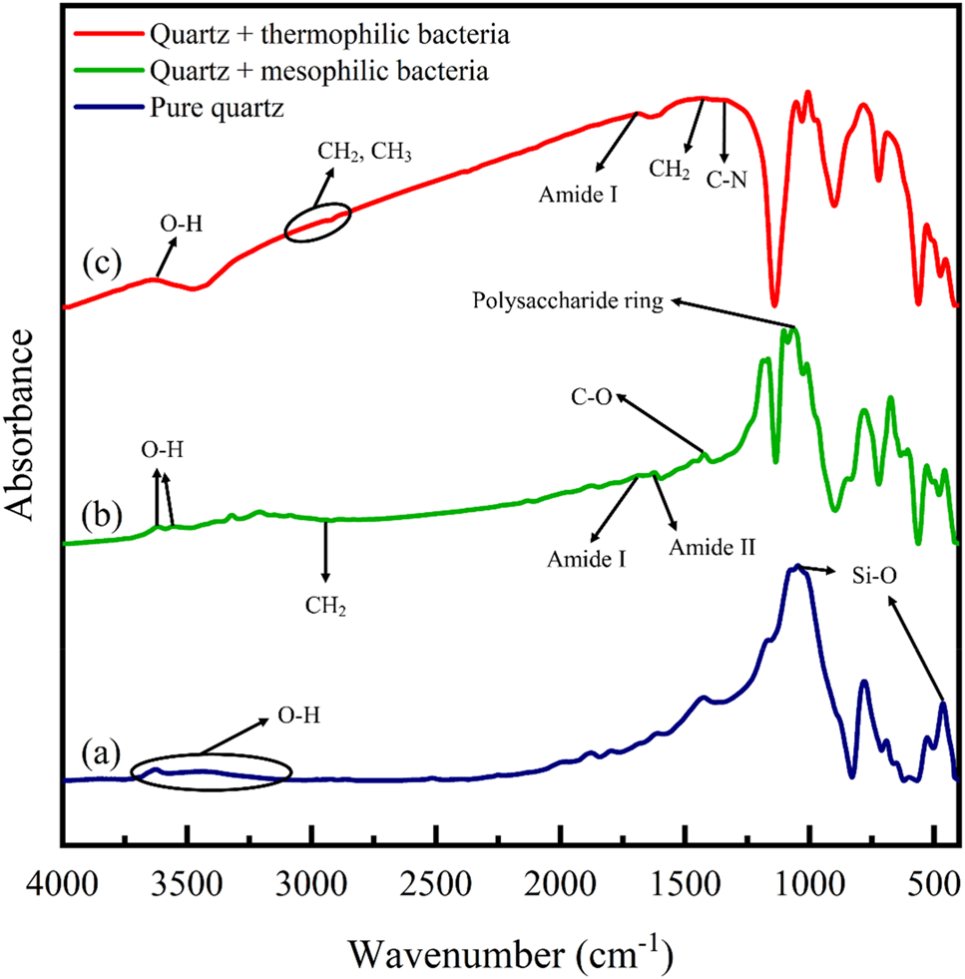
FTIR analysis of quartz adsorbent; (**a**) before aging, (**b**) after aging with mesophilic bacteria, (**c**) after aging with moderate thermophilic bacteria.

**Figure 5:**
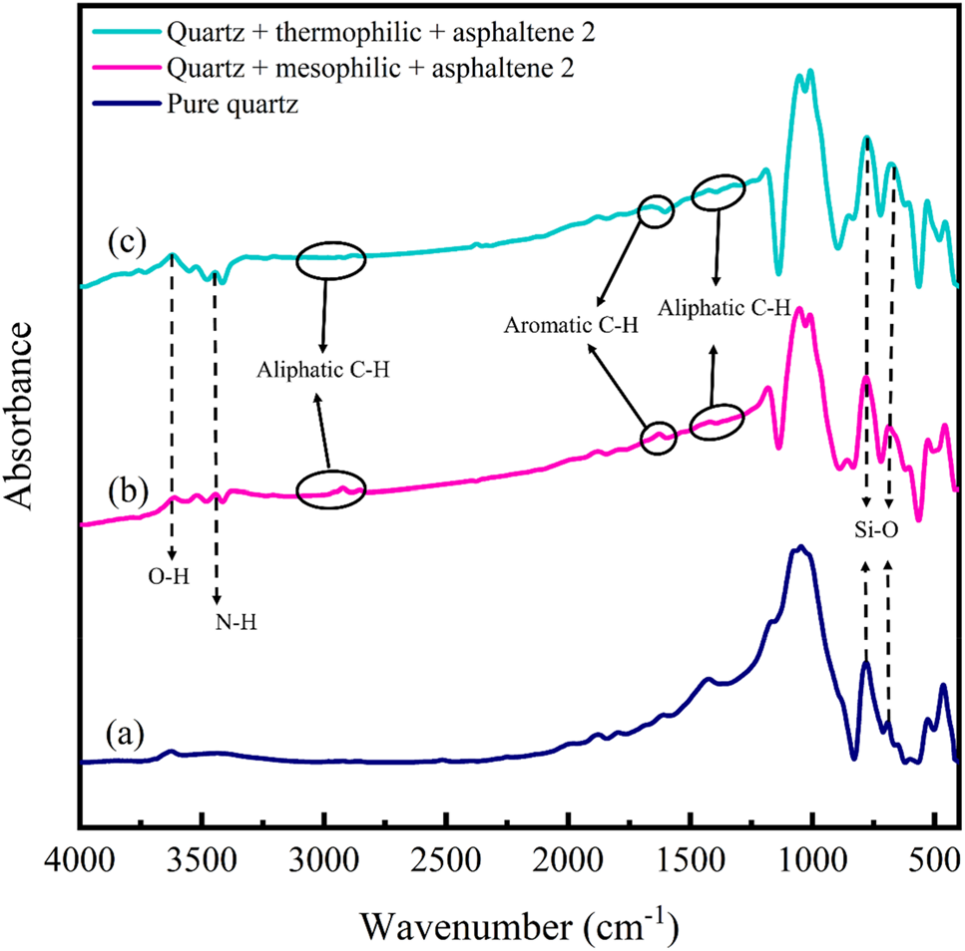
FTIR analysis of quartz adsorbent, (**a**) before asphaltene adsorption, (**b**) after asphaltene 2 adsorption on the adsorbent aged withmesophilic bacteria, (**c**) after asphaltene 2 adsorption on the adsorbent aged with moderate thermophilic bacteria.

**Figure 6:**
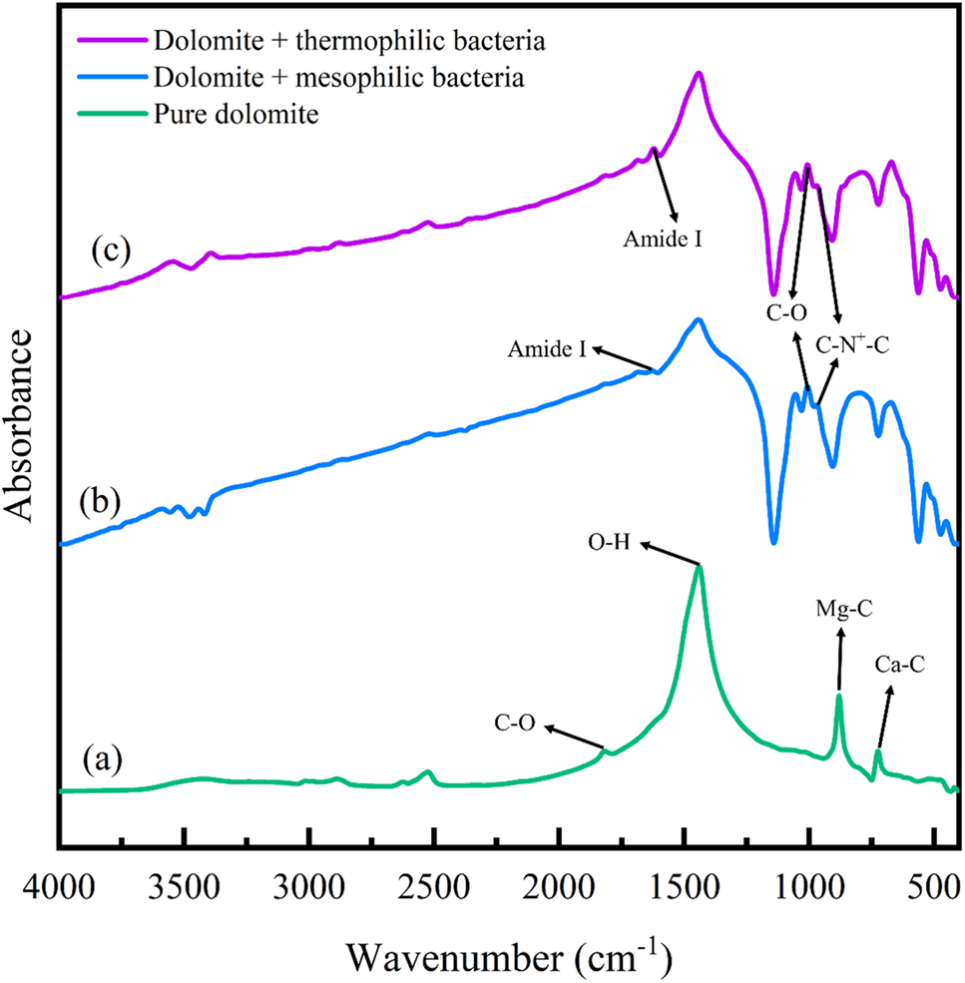
FTIR analysis of dolomite adsorbent, (**a**) before aging, (**b**) aging with mesophilic bacteria, (**c**) aging with moderate thermophilic bacteria.

**Figure 7:**
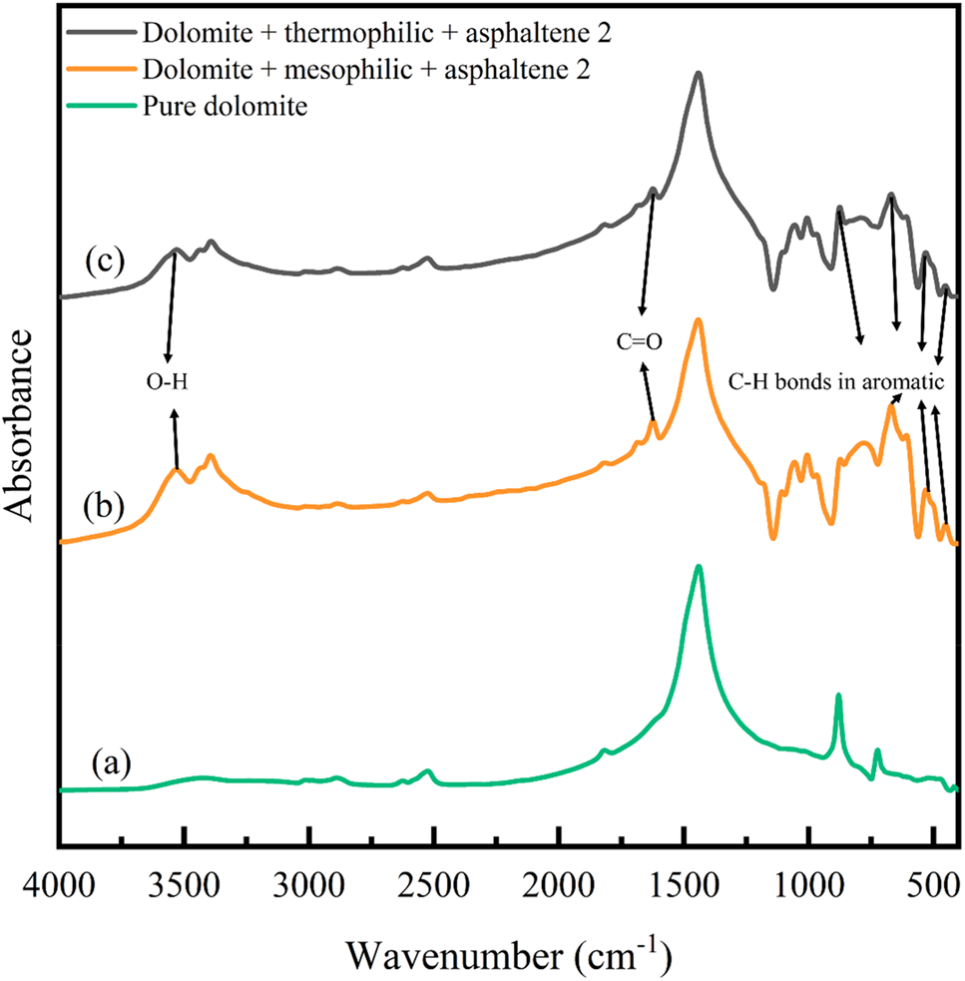
FTIR analysis of dolomite adsorbent, (**a**) before asphaltene adsorption, (**b**) after asphaltene 2 adsorption on the adsorbent aged with mesophilic bacteria, (**c**) after asphaltene 2 adsorption on the adsorbent aged with moderate thermophilic bacteria.

FTIR analysis at 400 and 4000 cm^−1^ wavenumbers was utilized to identify functional groups. Figures 4, 5, 6, 7 show the FTIR spectra of quartz and dolomite adsorbents before adsorption, after aging in bacteria, and after asphaltene adsorption.

#### Investigating the effect of bacteria on quartz by FTIR analysis

The Fig. 4a indicates the quartz adsorbent before aging in bacteria. The spectral range from 400–1200 cm^−1^ is called the fingerprint region. Four bonds are observed in this region. The wavenumber of 464 and 1075 cm^−1^ indicate the asymmetric stretching and bending vibration of Si–O. The wavenumber of 778 and 691 cm^−1^ indicate the symmetric stretching and bending vibrations of Si–O^76–78^. The 3100–3700 cm^−1^ wavenumber exhibits the stretching vibration of phenol O–H bonds^19^. The peak of O–H bond for quartz was observed at the wavenumber^76^ of 3622 cm^−1^. Figure 4b illustrates the quartz adsorbent after aging in mesophilic bacteria. New peaks have emerged in this part compared to the quartz before aging. The wavenumber 1065 cm^−1^ is the vibrations of the polysaccharide ring, and the wavenumber 1423 cm^−1^ is related to the C–O bending bond of carboxylate. Also, 1624 cm^−1^ wavenumber is the amid II vibrations, N–H or C–N of the protein. The wavenumber is 1683 cm^−1^ of the amide Ι (CO) bond. The wavenumber 2945 cm^−1^ shows the asymmetric CH_2_ stretching vibration of the fatty acid. The 3555 and 3616 cm^−1^ wavenumbers correspond to the symmetric stretching vibration of water molecules. The 458 and 529 cm^−1^ wavenumber are related to Si–O–Si bending vibration, and strong bonds in the approximate wavenumber of 1011, 1067, 1103, 1167, and 1185 cm^−1^ are related to Si–O stretching vibrations^19, 79^. After the adsorption of bacteria and the influence of biosurfactants, the vibrations of water molecules adsorbed on quartz altered from wavenumber 3622 to 3616 cm^−1^. These changes indicate that water molecules play an essential role in the adsorption of bacteria on quartz^79^. Figure 4c demonstrates the quartz adsorbent after aging in moderate thermophilic bacteria. New broad peaks have emerged in the fingerprint region compared to quartz before aging. The wavenumber of 1686 cm^−1^ indicates the amide Ι bond^80^. Approximate wavenumber of 1373 cm^−1^ and 1430 cm^−1^ indicate the stretching of cytosine C–N and CH_2_ bond of polysaccharides^81^. The wavenumber of 3637 cm^−1^ is related to O–H bonds^19^. The 2956–2850 cm^−1^ wavenumbers are CH_2_ and CH_3_ stretch bonds associated with lipids, proteins, carbohydrates, and nucleic acids^82^.

#### Investigation of asphaltene adsorption on quartz by FTIR analysis

The Fig. 5a depicts the quartz adsorbent before asphaltene adsorption. Figure 5b, c indicate the adsorption of asphaltene 2 on quartz after the influence of mesophilic and moderate thermophilic bacteria, respectively. New peaks appeared after the asphaltene adsorption on quartz. The 778 and 691 cm^−1^ wavenumber illustrate the symmetric bending and stretching vibrations of Si–O for quartz, which decreased to 777 and 678 cm^−1^ after asphaltene adsorption. This reduction indicates a polar interaction between asphaltene and hydroxyl on the quartz surface^19^. In the 3000–3700 cm^−1^ range, peaks associated with O–H or N–H stretching bonds were detected, and O–H stretching is broader and more intense than N–H due to being more electronegative than nitrogen. The wavenumber 3611 cm^−1^ shows the O–H bond, which with the addition of the asphaltene molecule and the creation of a hydrogen bond between the two molecules, leads to a decrease in the intensity of the free O–H stretching peak^5, 19^. Aliphatic C–H bonds were observed at (2900–3100 cm^−1^), and (1350–1500 cm^−1^), and aromatic C–H bonds were detected at (1600–1700 cm^−1^)^83^. The 3443 and 3208 cm^−1^ wavenumber indicate free pyrrolic NH and hydrogen bonds, respectively^5^. The aliphatic peaks at (2900–3100 cm^−1^) and (1350–1500 cm^−1^) for the adsorption of asphaltene on quartz after the influence of bacteria are weak, while the aromatic peak at (1600–1700 cm^−1^) is a little stronger. The phenomenon is linked to the expansion of hydrogen bonding on the surface of the adsorbent, prompted by the shift toward hydrophilicity. This expansion can potentially increase the availability of hydrogen bonding sites, thereby enhancing the hydrogen bonding capacity of molecules. Consequently, the observed weaker aliphatic peaks at (2900–3100 cm^−1^) and (1350–1500 cm^−1^) could arise from changes in hydrogen bonding interactions influencing the orientation or conformation of aliphatic chains. In contrast, a slightly more intense aromatic peak at (1600–1700 cm^−1^) can be attributed to heightened hydrogen bonding interactions that influence the molecular environment around aromatic groups and impact the C = O stretching vibration. The unusual inversion in the intensity of overtone peaks of aromatic C–H bonds at 1600 cm^−1^ may result from distinct conformational changes induced by the introduced polar interactions. In a similar condition observed in a study concerning the inhibition of asphaltene precipitation by TiO_2_ nanoparticles^83^, the presence of TiO_2_ nanoparticles within the oil under acidic conditions induces structural alterations in the asphaltene components. This modification renders the asphaltene system more stable, subsequently resulting in the attenuation of aliphatic peaks' intensity and a subtle enhancement in the aromatic peak's strength^83^.

#### Investigating the effect of bacteria on dolomite adsorbent by FTIR analysis

The Fig. 6a illustrates dolomite adsorbent before aging with bacteria. This figure indicates that 728 and 878 cm^−1^ wavenumbers correspond to the stretching vibration for Ca–C and Mg–C bonds. Also, 1094 and 1817 cm^−1^ wavenumbers show the stretching vibration of the C–O carboxylic bond. The wavenumber 1440 cm^−1^ is related to the bending vibration of O–H bonds. The 3100–3400 cm^−1^ wavenumbers indicate the stretching vibration of phenol O–H bonds, which is related to the adsorption of water molecules on the rock surface^84, 85^. The spectra range of 650–900 cm^−1^ is called the low-frequency region. The presence of broad and slightly sharp peaks indicates aromaticity, compounds of carboxylic acid dimers, amines, and amides in the molecular structure^86^. Figure 6b,c indicate dolomite adsorbent after aging in mesophilic and moderate thermophilic bacteria. In this figure, new peaks have appeared compared to the pre-aged dolomite. Upon the influence of moderate thermophilic bacteria on dolomite, a new peak emerged at the wavenumber of 969 cm^−1^, indicating C–N^+^–C stretching of nucleic acids^87^. Also, C–O oligosaccharide bonds were detected at the wavenumber of 1055 cm^−1^, which may interact in the hydroxyl group with other membrane components, including phosphate of phospholipids, phosphate ester stretch, and C–OH stretch^81^. The wavenumber 1441 cm^−1^ shows the lipid CH_2_ bending bond and 1685 cm^−1^ shows the amide Ι bond. The wavenumber in the 2800–3000 cm^−1^ region indicates the symmetric and asymmetric stretch bonds of CH_3_ and CH_2_. The wavenumber in the 3000–3700 cm^−1^ region is the stretching bonds of O–H and N–H related to water, protein, and polysaccharides^81, 87^. In the case of the influence of mesophilic bacteria on dolomite, the wavenumber is almost the same and is close to the wavenumber of moderate thermophilic bacteria; Only there is an amide I bond at the newly appeared wavenumber^81^ of 1639 cm^−1^.

#### Investigation of asphaltene adsorption on dolomite adsorbent by FTIR analysis

The Fig. 7a,b,c indicates the dolomite adsorbent before adsorption and after asphaltene adsorption on the adsorbent that was previously aged with mesophilic and moderately thermophilic bacteria. After the asphaltene adsorption, new peaks with approximately the same wavenumber appeared for (b) and (c) parts. The 400–1000 cm^−1^ is called the aromatic region and corresponds to the C–H bonds in aromatic rings. In the wavenumber spectrums of 1000–1600 cm^−1^, there are various bonds, such as C–O, C–N, C–C (single bonds), and bending bonds. The 1600–2000 cm^−1^ spectrums are known as the stretching region of the double bond because C = C, C = N, and C = O bonds are found in this region. The 2000–2300 cm^−1^ spectra are called the triple bond stretching region, which includes C≡C and C≡N bonds. The 2500–3700 cm^−1^ spectra are the stretching region of hydrogen because C–H, N–H, and O–H vibrations occur in this area^10^.

### The effect of the quantity of adsorbents on the adsorption of asphaltenes

**Figure 8:**
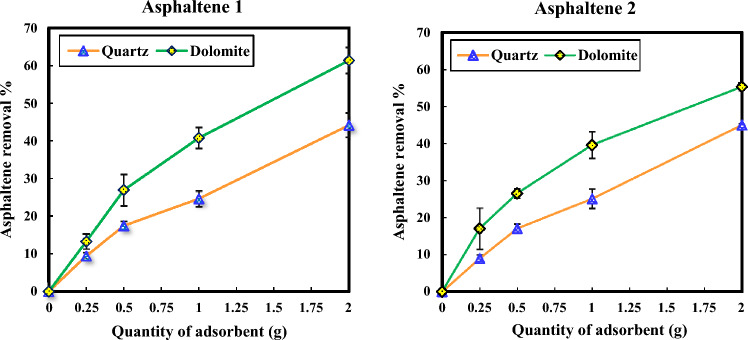
Investigating the effect of the amount of quartz and dolomite adsorbents on asphaltene removal efficiency for asphaltenes 1 (left) and 2 (right) at a concentration of 200 ppm.

The removal percentage of asphaltenes 1 and 2 are illustrated in Fig. 8. As can be seen, the asphaltene removal efficiency extends with the increase in the micro adsorbent mass. The increase in removal efficiency can be attributed to the expansion of available adsorption sites achieved by increasing the mass of the micro adsorbent. However, this growth follows a nonlinear pattern, as certain adsorption sites might remain unsaturated or unoccupied throughout the adsorption process^17^. The adsorption of asphaltenes can be influenced by a range of factors including temperature, the type of precipitant, the presence of water, and the composition of asphaltene compounds. The asphaltene characteristics, such as the content of heteroatoms, chemical groups, and aromatic nature, play a significant role in determining the extent of asphaltene adsorption^88^. Increasing the aromaticity of asphaltenes (low molecular weight asphaltenes and low C/H ratio) and high nitrogen content enhance the self-aggregation of asphaltenes, increasing the interaction between asphaltenes and adsorbents^13, 18^. The results of the asphaltenes adsorption on quartz and dolomite show that the adsorption of two asphaltenes is nearly identical to each other. In some cases, the adsorption of asphaltene 2 is relatively higher. According to Table 2, which indicates the elemental analysis results, asphaltene 2 has a low H/C ratio (more aromatic rings) and more nitrogen content, which can be the reason for the more uptake of this asphaltene by the adsorbents. However, in the case of dolomite, the final extent of adsorption achieved by asphaltene 1 surpasses that of asphaltene 2. This disparity in adsorption behavior between the two types of asphaltenes could arise from a combination of factors related to the inherent characteristics of each asphaltene type and the unique interaction mechanisms between these compounds and the dolomite surface. Moreover, the topography and charge distribution of the dolomite surface could play a role in the differential adsorption behavior. This variation in surface affinity could account for the observed difference in final adsorption levels. To draw a comprehensive inference regarding the diverse adsorption tendencies of various asphaltenes on dolomite, an in-depth exploration encompassing a large number of asphaltenes and their corresponding concentrations necessitates further investigation. This avenue, however, lies beyond the current scope of our study and has already undergone thorough examination within existing literature^38^. Our inquiry underscores that the impact of the asphaltenes' total nitrogen and sulfur content, interwoven with their intricate aromatic features, emerges as the foremost factors shaping the process of asphaltene adsorption onto the dolomite^38^. Figure 8 shows that asphaltenes adsorption on the dolomite micro-adsorbent is higher than that of the quartz micro-adsorbent. The higher metal content of dolomite adsorbent compared to quartz adsorbent can be one of the reasons for its higher asphaltene adsorption^38^.

### The effect of bacteria on the static adsorption of asphaltenes

**Figure 9:**
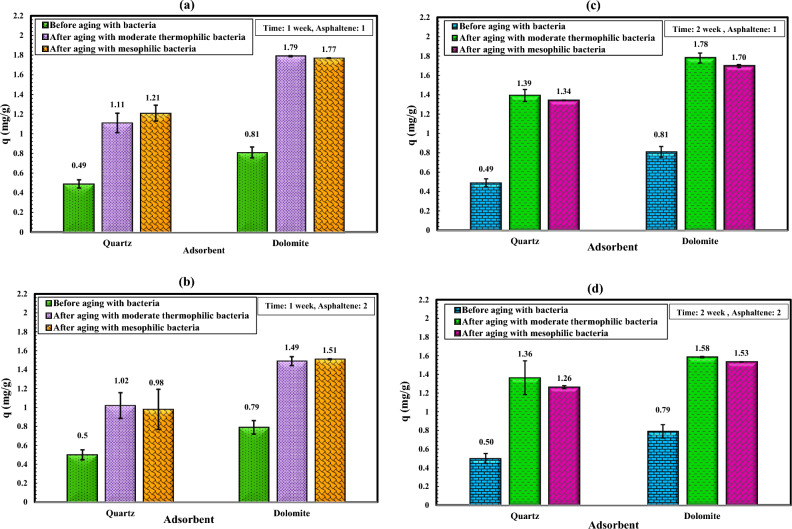
Effect of moderate thermophilic and mesophilic bacteria on the asphaltenes adsorption on the quartz and dolomite adsorbents at different intervals (**a** and **b** one week) (**c** and **d** two weeks).

The Fig. 9 depicts the influence of moderate thermophilic and mesophilic bacteria at different times: one week (Fig. 9a,b) and two weeks (Fig. 9c,d), on the static adsorption of asphaltenes 1 and 2 on quartz and dolomite adsorbents. Figure 9 shows that after two weeks of aging of adsorbents in moderate thermophilic bacteria, the adsorption of asphaltene 1 on quartz increased from 0.49 to 1.39 mg/g, and the adsorption increased from 0.81 to 1.78 mg/g for dolomite. Moreover, for mesophilic bacteria, the adsorption of asphaltene 1 on quartz increased from 0.49 to 1.34 mg/g, and the adsorption increased from 0.81 to 1.70 mg/g for dolomite. The adsorption amount of asphaltene 2 after the impact of moderate thermophilic bacteria for quartz increased from 0.5 to 1.36 mg/g and for dolomite increased from 0.79 to 1.58 mg/g. Also, for mesophilic bacteria, the adsorption of asphaltene 2 on quartz increased from 0.5 to 1.26 mg/g, and for dolomite increased from 0.79 to 1.53 mg/g. In contrast to the previous scenario without bacterial effects (Fig. 8), in the experiments following the bacterial influence, asphaltene 1 shows higher adsorption on the adsorbents compared to asphaltene 2 (Fig. 9). According to Table 2, asphaltene 1 exhibits greater polarity, possibly stemming from its composition or the presence of polar functional groups. This increased polarity fosters stronger polar interactions, such as hydrogen bonding, between asphaltene 1 and the modified adsorbent surfaces with bacterial solution. As FTIR analysis showed earlier, the expansion of hydrogen bonds on the surface, brought about by the shift towards hydrophilicity due to biosurfactant production, enhances the affinity and adhesion of asphaltene 1 to the adsorbent. Conversely, asphaltene 2, with comparatively lower polarity, may have weaker polar interactions with the modified surfaces. The reduced affinity might result from a diminished presence of polar functional groups, thereby leading to lesser adsorption onto the modified adsorbents.

The essential mechanisms ruling the adsorption of bacteria-minerals still need to be better understood. The adsorption of bacteria on mineral surfaces is driven by both electrostatic and non-electrostatic interactions. The electrostatic force originates from the coulombic interaction between two charged entities, while the non-electrostatic force is a result of various interactions such as hydrogen bonding, van der Waals forces, hydrophobic interactions, etc.^79^. The literature review on the adsorption of bacteria on different surfaces showed that the adsorption behavior of bacteria is sensitive to changes in the ionic strength of the solution, mineralogy, pH, and bacterial concentration. The adsorption of bacteria strongly depends on pH and decreases significantly with increasing pH^89^. High negative zeta potential is equated with hydrophilicity, while low negative zeta potential values and positive zeta potential values are associated with hydrophobicity^90^. Increased bacterial adsorption occurs when bacterial and mineral surfaces exhibit opposite surface charges, with decreased adsorption as surface charges change and as pH increases, both charges become negative^89^. Generally, negatively charged bacteria easily adhere to positively charged surfaces through electrostatic attraction^91^. Due to the tendency of carbonate rocks, including dolomite, to have positively charged surfaces under different pH values of the formation^41^, especially in the low pH of this bacterial medium, dolomite has a high positive charge compared to sandstone minerals (usually negatively charged), so one of the factors that the increase in asphaltene adsorption on dolomite compared to quartz is the effect of its surface charge. A literature review indicated that the adsorption of bacteria to minerals coated with iron or comprising more amounts of iron significantly increased bacterial adhesion^79, 89^. According to Table 1 of the XRF analysis, the iron content of the dolomite adsorbent is more than that of the quartz adsorbent. Therefore, another factor that improves asphaltene adsorption by dolomite adsorbents is the amount of iron contained therein, which increases the adhesion of bacterial cells. As a result, its surface has become more hydrophilic, and more hydrogen bonds have formed between dolomite and asphaltene. Figure 9 illustrates that bacteria significantly affected the surface of dolomite and quartz adsorbents during the first week of aging. When the aging time was increased from one week to two weeks, the adsorption of asphaltene onto the dolomite adsorbent did not change significantly, while the adsorption of asphaltene onto the quartz adsorbent improved. The observed phenomenon may be due to dolomite surface charge, which may result in an enhanced bacterial adhesion capacity and biosurfactant production during the initial phases of aging.

### Wettability alteration

#### Wettability of dolomite before aging

**Figure 10:**
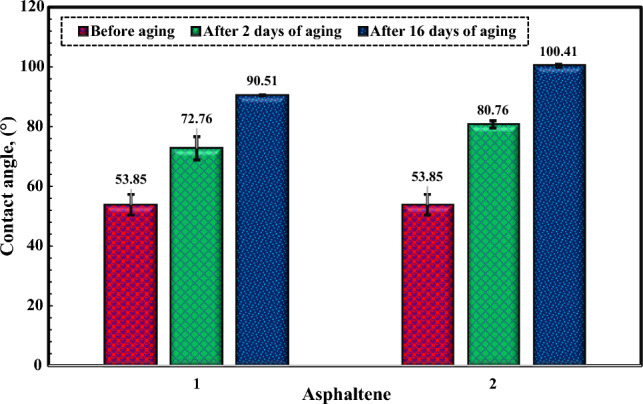
Contact angle changes of dolomite after aging in asphaltenes 1, 2, and at different times.

Based on Fig. 10, before undergoing any aging process, the contact angle measured for dolomite stood at 53.85°. This reading suggests that the samples exhibited water-wet characteristics at the outset.

#### The influence of asphaltene on the dolomite rock wettability

The Fig. 10 indicates how the contact angle for asphaltenes changes over time. The contact angle of the dolomite samples for asphaltenes 1 and 2 increased from 53.85° to 90.51° and 100.41°, respectively. As shown in Fig. 10, the aging time plays a crucial role in the wettability of mineral surfaces. As the contact time between asphaltene and minerals increased, the contact angle of water with dolomite increased, and the mineral became more oil-wet. Rock minerals and asphaltene molecules have an inherent net surface charge, and the two molecules must be oppositely charged to cause a change in wettability. Considering that the samples were dry-aged in asphaltene under these conditions, the interactions altering the surface wettability can be polar interactions between the functional groups. Asphaltene is one of the heaviest and most polar components of crude oil and has heteroatoms such as sulfur, nitrogen, oxygen, and some functional groups^9, 42, 49^. Due to the tendency of carbonate rocks, such as dolomite, to have a positively charged surface at different pH values of the formation^41^, the interaction of asphaltenes with the rock surface increases, and asphaltene is adsorbed to the rock surface, altering wettability^9^. Figure 10 indicates that the wettability alters of asphaltene 2 at the rock surface are more significant than that of asphaltene 1. According to the elemental results in Table 2, asphaltene 2 has more aromatic rings (low H/C ratio) and higher nitrogen content than asphaltene 1. The outcome is an amplified interaction between asphaltene and the adsorbent, consequently fostering increased adsorption of asphaltene onto dolomite. This heightened adsorption, in turn, instigates a more pronounced alteration in wettability, from an initial water-wet state towards intermediate wettability, eventually progressing to an oil-wet condition.

#### The influence of bacterial solution on the dolomite rock wettability

**Figure 11:**
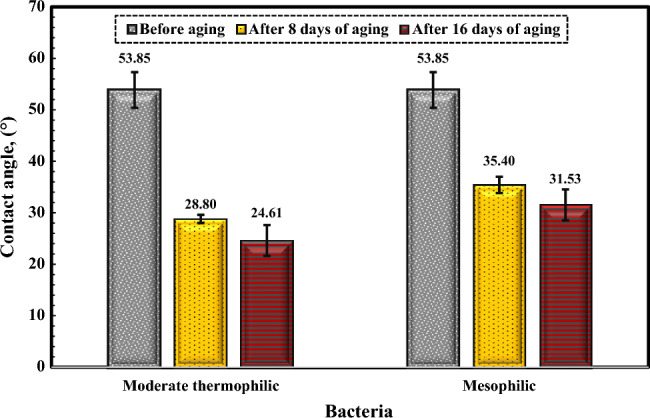
Contact angle changes of dolomite after aging in moderate thermophilic and mesophilic bacteria at different times.

The Fig. 11 shows the effect of moderately thermophilic and mesophilic bacteria and the changes in contact angle over time. After 16 days, the contact angle of the rock sample exhibited a decline from 53.85° to 24.61° when subjected to moderate thermophilic bacteria, and similarly, from 53.85° to 31.52° when exposed to mesophilic bacteria. The results indicate that the bacteria were most effective in the first week. The samples became extremely water-wet due to biosurfactant production or bacterial adhesion to the surfaces, with the noteworthy observation that the influence of moderate thermophilic bacteria on the wettability change of the dolomite surface was notably more substantial compared to mesophilic bacteria.

#### Influence of asphaltene on the dolomite wettability after aging in bacterial solution

**Figure 12:**
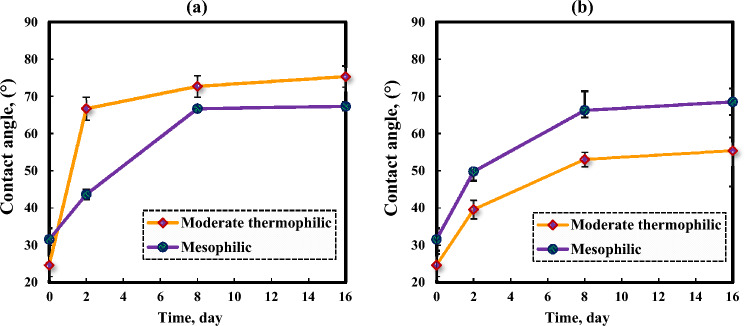
Contact angle changes of dolomite after aging in asphaltenes: (**a**) 1 and (**b**) 2 after the influence of moderate thermophilic and mesophilic bacteria at different times.

The Fig. 12 shows the changes in the contact angle of dolomite (pre-aged with bacteria) after aging in asphaltenes 1 and 2 at different times. After 16 days of dolomite aging in asphaltene 1, the contact angle for dolomite that formerly was aged with thermophilic bacteria increased from 24.61° to 75.30° and for moderate mesophile from 31.52° to 67.31°. The results indicate the considerable effect of moderate thermophilic bacteria on asphaltene 1 adsorption. Figure 12 shows that the influence of mesophilic bacteria on the wettability of asphaltene 2 is influential, and the contact angle increased from 31.52° to 68.53°. Also, for moderate thermophilic bacteria, the contact angle alters from 24.61° to 55.37° after aging in asphaltene 2. In general, the adsorption of asphaltene 1 on the dolomite and its wettability changes after the effect of bacteria is more than asphaltene 2.

#### Analysis and investigation of dolomite contact angle changes after aging

**Figure 13:**
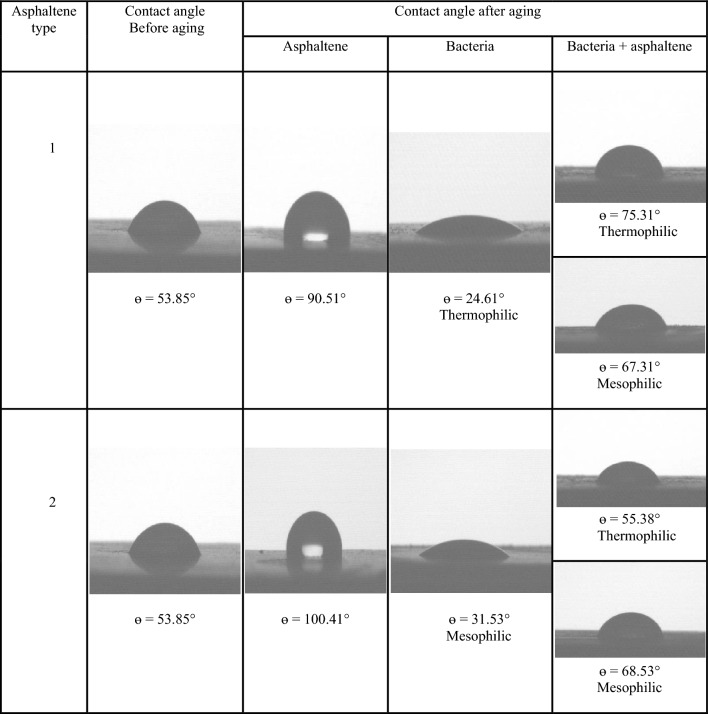
The contact angle of water droplets on dolomite before and after 16 days of aging.

**Figure 14:**
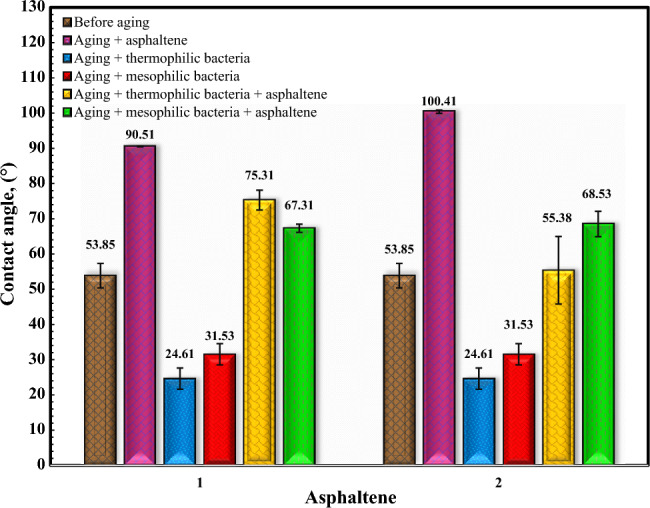
Comparison of dolomite contact angle for Asphaltenes 1 and 2 after 16 days of aging under different conditions.

The Fig. 13 illustrates the features of the contact angle of water droplets on dolomite before and after aging in asphaltene solutions, moderately thermophilic and mesophilic bacteria, and re-aging in asphaltene after the influence of bacteria after a contact time of 16 days. Figure 14 shows the comparison of the contact angle of dolomite before aging, after aging in asphaltenes, after aging in bacteria, and after aging of dolomite with asphaltenes (after the influence of bacteria on the dolomite surface) after 16 days. Figure 14 illustrates that dolomite samples were water-wet before aging and had a contact angle is 53.85°. After aging in asphaltenes, the contact angle for asphaltene 2 altered to 100.41° and for asphaltene 1 to 90.51°. As the samples were soaked dry in asphaltene, polar interaction was the primary mechanism of wettability change, and the samples became oil-wet. Considering that asphaltene 2 has an aromatic nature and more nitrogen content, it improves its interaction with the dolomite surface, and as a result, the adsorption of asphaltene on it increases, and finally, it becomes more oil-wet. After aging of the samples in the solution of moderate thermophilic and mesophilic bacteria, the contact angle decreased from 53.85° to 24.61° and 31.53°, respectively, and the samples became extremely water-wet, which is due to various mechanisms of wetting such as biosurfactant production and bacterial adhesion, on dolomite surfaces. Figure 14 indicates that the contact angle of the samples aged with moderate thermophilic bacteria has decreased more.

The bacterial cell wall consists of teichoic acids (for gram-positive bacteria), lipopolysaccharides (for gram-negative bacteria), surface proteins, and extracellular polymeric substances, which are a heterogeneous mixture of polysaccharides, proteins, nucleic acids, and lipids. The main reactive groups on the surface of the cell wall are the carboxyl and phosphate moieties^92^. Additional surface polysaccharides make the bacteria more hydrophilic, unlike additional protein compounds, which make surfaces relatively more hydrophobic^93^. FTIR analysis results about moderate thermophilic bacteria show that the peak intensity of CH_2_ asymmetric stretching vibration is higher than mesophilic, indicating a higher concentration of fatty acyl chains in moderate thermophilic bacteria. Higher amounts of amide I and amide II area support high protein content in moderate thermophilic bacteria. The amino acid composition of moderate thermophilic proteins is usually higher than mesophilic. The amino acid composition of proteins of mesophilic and thermophilic microorganisms usually reflects the mechanism of molecular adaptation to extreme physical conditions. The benefit of amino acid exchange, often reported in thermophilic proteins, is that more hydrogen bonds may be formed. The increase in the number of hydrogen bonds with the adsorbent improves the hydrophilicity of the adsorbent surface. Therefore, the connection between asphaltene and the adsorbent through hydrogen bonds increases, and as a result, the adsorption of asphaltene on the adsorbent increases and causes an additional change in wettability and increased oil-wetting^87^.

Finally, it should be noted that the primary contribution of this study underscores the dual impact of the used microorganisms. Notably, they play a critical role in reducing both the interfacial tension, as demonstrated in previous studies^57, 58^, and the contact angles of reservoir rocks, shifting wettability towards a strongly water-wet condition, as evidenced in this research. These alterations are crucial for enhancing oil recovery. While there is an observed increase in asphaltene adsorption on reservoir rock minerals, potentially posing challenges such as formation damage, particularly in low-permeability reservoirs, the findings emphasize that the positive effects of bacteria overwhelmingly prevail. This clarification aims to address the question of the real-life implications and underline the overall value of this manuscript, particularly for individuals working in the oil and gas industry.

In the end, it is noteworthy to acknowledge that the intricate interplay between microorganisms, minerals, and asphaltene interactions is poised at the forefront of research endeavors within the realm of rock wettability alteration, asphaltene-related phenomena, and eventually MEOR processes. The intricacy inherent in these interactions underscores the imperative for comprehensive and rigorous investigations in future research endeavors that encompass various facets. These facets include examining the impact of bacterial solutions on rock wettability after aging in asphaltene, exploring the use of anaerobic microorganisms in an anaerobic environment to simulate reservoir conditions, and the simultaneous scrutiny of asphaltene adsorption alongside the presence and growth of microorganisms on adsorbents. As we advance towards a more holistic comprehension of these phenomena, our ability to harness their potential for optimizing oil recovery and reservoir management gains renewed impetus. This pursuit not only augments our scientific understanding but also holds promise for enhancing the efficiency and sustainability of oil recovery strategies in the ever-evolving landscape of petroleum engineering.

## Conclusions

In this study, the effect of bacteria on the adsorption of asphaltenes extracted from two oil samples with different origins on micro-sized quartz and dolomite minerals was studied. Moreover, the effect of bacteria and asphaltenes on dolomite surface wettability was investigated. The main results of this research are as follows:

1. After aging with mesophilic bacteria, the quartz adsorbent exhibits new peaks in its FTIR spectrum, including vibrations related to polysaccharide rings (1065 cm^−1^), carboxylate C-O bending (1423 cm^−1^), amid II vibrations (1624 cm^−1^), and amide I (CO) bonds (1683 cm^−1^). Moderate thermophilic bacteria aging on the quartz adsorbent induces the appearance of new broad peaks in its FTIR spectrum, highlighting features like the amide I bond (1686 cm^−1^), cytosine C–N stretching (1373 cm^−1^), and CH_2_ bond of polysaccharides (1430 cm^−1^). Additionally, O–H bonds (3637 cm^−1^) and CH_2_/CH_3_ stretches (2956–2850 cm^−1^) associated with lipids, proteins, carbohydrates, and nucleic acids were identified.
2. After mesophilic and moderate thermophilic bacterial exposure, the dolomite adsorbent exhibits new peaks. Under moderate thermophilic influence, a peak at 969 cm^−1^ signifies C–N^+^–C nucleic acid stretching, while 1055 cm^−1^ indicates C–O oligosaccharide bonds interacting with hydroxyl groups and membrane components. Peaks at 1441 cm^−1^ (lipid CH_2_ bending), 1685 cm^−1^ (amide Ι bond), and 3000–3700 cm^−1^ (O–H and N–H stretching related to water, protein, and polysaccharides) are consistent with both bacterial influences, with mesophilic exposure introducing an additional amide I bond at 1639 cm^−1^.
3. FTIR analysis for quartz and dolomite rocks demonstrated that after aging in bacterial solution, bonds related to the adsorption of bacterial cells and biosurfactant production appear, which are the main factors of change in wettability. Alteration in wettability towards hydrophilicity expands hydrogen bonds on the surface, thus improving asphaltene adsorption due to polar interaction.
4. After two weeks of aging of minerals in moderate thermophilic bacteria, the adsorption of asphaltene 1 on quartz increased from 0.49 to 1.39 mg/g, and the adsorption increased from 0.81 to 1.78 mg/g for dolomite. Moreover, for mesophilic bacteria, the adsorption of asphaltene 1 on quartz increased from 0.49 to 1.34 mg/g, and the adsorption increased from 0.81 to 1.70 mg/g for dolomite. The adsorption amount of asphaltene 2 after the impact of moderate thermophilic bacteria for quartz increased from 0.5 to 1.36 mg/g and for dolomite increased from 0.79 to 1.58 mg/g. Also, for mesophilic bacteria, the adsorption of asphaltene 2 on quartz increased from 0.5 to 1.26 mg/g, and for dolomite increased from 0.79 to 1.53 mg/g. Overall, the results indicated that after two weeks of mineral aging in moderate thermophilic bacteria, the adsorption of asphaltene on both minerals increased between 180 and 290%.
5. Asphaltene 1 altered the contact angle of dolomite from 53.85° to 90.51° and asphaltene 2 from 53.85° to 100.41°. Adsorption of asphaltenes on dolomite rocks that formerly were aged with bacteria indicated that bacteria retain wettability in a hydrophilic state.
6. The effect of moderate thermophilic bacteria on dolomite surface wettability is higher than mesophilic bacteria due to the more protein content of this bacteria, which increases hydrogen bonding with the surface. After 16 days, the contact angle of the rock sample decreased from 53.85° to 24.61° when exposed to moderate thermophilic bacteria and similarly, from 53.85° to 31.52° when exposed to mesophilic bacteria, with the bacteria being most effective in the first week of exposure. The samples became extremely water-wet due to biosurfactant production or bacterial adhesion to the surfaces.

In conclusion, this study emphasizes the dual influence of the utilized microorganisms. Firstly, they markedly decrease contact angles, promoting a highly water-wet condition that is beneficial for MEOR. However, these microorganisms can also enhance asphaltene adsorption on reservoir rock minerals, presenting a potential issue in the form of formation damage, especially in low-permeability reservoirs.

## Data Availability

The databank utilized during this research is available from the corresponding author on reasonable request.
